# Association of urinary eosinophilic protein X at age 3 years and subsequent persistence of wheezing and asthma diagnosis in adolescence

**DOI:** 10.1111/pai.70013

**Published:** 2024-12-04

**Authors:** Iso Precious Oloyede, Anhar Ullah, Clare S. Murray, Sara Fontanella, Angela Simpson, Adnan Custovic

**Affiliations:** ^1^ National Heart and Lung Institute Imperial College London London UK; ^2^ Division of Infection, Immunity and Respiratory Medicine, Faculty of Biology, Medicine and Health, Manchester Academic Health Sciences Centre University of Manchester and University Hospital of South Manchester NHS Foundation Trust Manchester UK

**Keywords:** asthma prediction, preschool wheeze, urinary eosinophilic protein X, wheeze phenotypes

## Abstract

**Background:**

Wheezing is common in early life, but most children stop wheezing by school age. However, the prediction of course of wheezing through childhood is difficult.

**Objective:**

To investigate whether urinary EPX (a marker of eosinophil activation) in children at age 3 years may be useful for the prediction of wheeze persistence and future asthma diagnosis.

**Methods:**

U‐EPX was measured at age 3 years (radioimmunoassay) in 906 participants in the population‐based birth cohort. Children attended follow‐ups to age 16 years. We investigate the discriminative ability of u‐EPX and other factors in predicting asthma diagnosis at age 16 using receiver operating characteristic [ROC] curves.

**Results:**

Of 613 children with follow‐up information at age 16, 511 had data on u‐EPX at age 3 and asthma diagnosis at age 16 years; of those; 133 (21.7%) had asthma. Based on longitudinal data, children were assigned to wheeze clusters: No wheeze (NWZ), early transient (ETW), late‐onset (LOW), intermittent (INT) and persistent wheeze (PEW). U‐EPX levels differed significantly between different wheeze clusters (*p* = .003), with clusters characterised with persistent symptoms having higher u‐EPX. In the whole cohort, the best performing classification model for asthma diagnosis at age 16 years included sex, u‐EPX, sensitisation and wheeze (area under the curve (AUC) = 0.82, 95% CI: 0.76–0.88). u‐EPX and allergic sensitisation alone had similar predictive power (AUC [95%CI]: 0.64 [0.58–0.71] and 0.65 [0.60–0.71]). The best performing classification model for asthma prediction among children with doctor‐confirmed wheeze in the first 3 years included child's u‐EPX and sensitisation at age 3 years, sex, gestational age and maternal atopy (AUC: 0.76, 95%CI: 0.67–0.85).

**Conclusions:**

Early‐life u‐EPX may be a useful non‐invasive marker for asthma prediction in adolescence.

**Abbreviations**
EPX: eosinophilic protein X
ETW: early transient wheeze
F1: harmonic mean of precision and recall
HDM: house dust mite
INT: intermittent wheeze
LOW: late‐onset wheeze
MAAS: Manchester Asthma and Allergy Study
ML: machine learning
NPV: negative predictive value
NWZ: no wheeze
OR: odds ratio
PAM: partition‐around‐medoids
PEW: persistent wheeze
PPV: positive predictive value
SPT: skin prick test
u‐EPX: urinary eosinophilic protein X creatinine ratio
+LR: positive likelihood ratio
−LR: negative likelihood ratio
95% CI: 95% confidence intervals

**Key message**

Urinary EPX, which can be measured non‐invasively in early childhood, may be a useful biomarker for the prediction of asthma diagnosis in adolescence. It is likely that such cost‐effective test measured in easily obtainable urine samples would be more acceptable to parents compared to more invasive and more expensive tests such as skin tests or blood IgEs.

## 1 INTRODUCTION

Preschool wheeze is common,^1^ but in most children it is transient and remits by school age (so called ‘transient wheeze’).^2^ In contrast, in others the symptoms persist and may subsequently be diagnosed as asthma (‘persistent wheeze’).^1^, ^2^, ^3^ However, at symptom onset, clinical presentation among children with transient and persistent wheeze is similar, thereby making the prediction of the course of wheezing through childhood in individual patients difficult.^4^ These uncertainties surrounding the diagnosis and prognosis of preschool wheeze have a major adverse impact on parents/carers of children with preschool wheeze, and accurate prognosis remains one of the key areas of unmet need where more information is needed from healthcare professionals.^1^

Various approaches have been used to predict the persistence of wheeze and subsequent diagnosis of childhood asthma, including clinical asthma prediction tools,^5^, ^6^, ^7^ machine learning (ML) models,^8^, ^9^, ^10^ lung function indices,^11^ markers of atopy^12^ and biomarkers in body fluids,^13^ with relatively limited clinical utility.^1^, ^14^ Some limitations include inability to generalise the findings, as well as the invasiveness, cost and availability of the various tests.^15^ For example, ML approaches have been shown to have better performance over the regression‐based models for childhood asthma prediction,^8^ but are not sufficiently accurate for routine use in clinical practice.^16^ Furthermore, integration of genomic risk scores (polygenic and methylation risk scores) only marginally improves the performance of such ML prediction model.^9^ Therefore, the search for a suitable non‐invasive biomarker for the prediction of wheeze persistence and/or subsequent development of asthma remains important.

The role of eosinophilic inflammation as a pathophysiologic mechanism in the development of asthma is well‐established.^17^ The activated eosinophils produces granule proteins that can be measured as biomarkers of eosinophilic inflammation. For example, eosinophil protein X (EPX) can be measured non‐invasively in urine and has a good correlation with activated eosinophils.^18^ These properties could therefore make it a potentially useful tool for the prediction of wheeze persistence or subsequent asthma diagnosis in routine paediatric practice.

We aimed to determine whether the urinary EPX levels at age 3 years differ in different longitudinal childhood wheeze phenotypes and investigate the relationship between urinary EPX at age 3 years and current asthma diagnosis in adolescence.

## 2 METHODS

### 2.1 Study design, setting, participants and data sources

The Manchester Asthma and Allergy Study (MAAS) is a population‐based birth cohort.^19^ The study was approved by the local research ethics committee and written informed consent was obtained from parents; study subjects gave their assent when applicable. Participants attended clinical follow‐ups at ages 1, 3, 5, 8, 11 and 16 years. Validated questionnaires were interviewer‐administered to collect information on symptoms, physician‐diagnosed illnesses and medication usage.

#### 2.1.1 Primary care medical records data

Data from the electronic and paper‐based primary care records were transcribed by a trained paediatrician,^20^ including the presence of wheeze, asthma diagnosis and therapy (inhaled corticosteroids and β2 agonist), unscheduled visits and hospital admissions for asthma/wheeze. These data were used to identify episodes of wheeze confirmed by the primary care physicians for each year up to 8 years of age.

#### 2.1.2 Skin prick testing

At age 3 years, skin prick testing was undertaken to house dust mite, cat, dogs, grass, moulds, milk, egg and peanut with negative and positive (histamine hydrochloride 10 mg/mL) controls (Bayer, Elkhart, IN, USA).

#### 2.1.3 Measurement of Urinary Eosinophilic protein X

Urine was collected at the age 3 years, divided in aliquots and frozen at −20^o^C.^21^ EPX was analysed in duplicate by means of a sensitive and specific radioimmunoassay (RIA) (Phadia AB, Uppsala Sweden). Due to the absence of the total urine volume, the extent of dilution of urine in the kidneys was determined by measuring the urine creatinine (Cr) using the alkaline picrate method (Jaffé reaction; HiCo Creatinine; Boehringer Mannheim GmbH, Mannheim, Germany). The results are presented as the EPX/Cr ratio (in micrograms per millimole [μg/mmol]; u‐EPX).^21^, ^22^

### 2.2 Definition of variables

#### 2.2.1 Current wheeze

A positive answer to the question ‘Has your child had wheezing or whistling in the chest in the last 12 months?’ at each of the clinical follow‐ups.

#### 2.2.2 Physician confirmed wheeze age 3 years

Defined using the data extracted from the primary care medical records as presence of wheeze confirmed by a general practitioner (GP) up to age 3 years (GP wheeze).

#### 2.2.3 Severe asthma/wheeze exacerbation

Defined from medical records data as admission to hospital or emergency department visits due to acute episode of wheezing/asthma and/or receipt of systemic corticosteroids for at least 3 days.^23^

#### 2.2.4 Allergic sensitisation

Wheal diameter of at least 3 mm greater than that elicited by the negative control to at least one of the tested allergens.

#### 2.2.5 Current Asthma at age 16 years

Defined as a positive response to any two of the following three questions: (1) ‘Has a doctor ever told you that your child has or had asthma?’ (2) ‘Has your child had wheezing or whistling in the chest in the last 12 months?’ (3) ‘In the past 12 months has your child used any medication for wheezing or asthma?’^24^

#### 2.2.6 Wheeze phenotypes (clusters)^25^

Recently, we developed a data‐driven method which for the assignment to wheeze clusters (phenotypes).^25^ Through clustering of six variables of wheezing spells (including duration, temporal sequencing and extent of persistence/recurrence), we derived five clusters which were internally homogenous and stable: (1) never wheeze (NWZ); (2) early transient wheeze (ETW); (3) late‐onset wheeze (LOW); (4) persistent wheeze (PEW); and (5) intermittent (INT) wheeze.^25^

### 2.3 Statistical analysis

Continuous variables were reported as mean and standard deviation (SD) or geometric mean and geometric standard deviation (SD). Categorical variables were reported as numbers and percentages. To study the association between u‐EPX and categorical variables, we used analysis of variance (ANOVA) or independent sample *t*‐tests where appropriate. Bonferroni correction was used to account for the multiple comparisons. To study the association between categorical variables, we used the chi‐squared test. The univariate and adjusted odds ratios with 95% confidence intervals (CI) were estimated using logistic regression. To investigate the discriminative ability of u‐EPX and other factors in predicting asthma diagnosis at age 16, we used receiver operating characteristic [ROC] curves. The results were reported as AUC (area under the curve). For u‐EPX an optimal cut off point was chosen using the Youden Index [YI].^26^ All analyses were performed using SAS 9.4 and R 4.2.2.

## 3 RESULTS

### 3.1 Characteristics of the study population

Figure S1 outlines the participant flow. Of 1184 children who were born into the cohort, 905 had data for u‐EPX at age 3 years, 613 attended follow‐up at age 16 and 511 had data for u‐EPX at age 3 and asthma diagnosis at age 16 years. Table S1 summarises the demographic and clinical characteristics of the study population; 36.77% (332/903) participants had parentally reported wheezing in the first 3 years of life, 40.98% (293/715) had doctor‐confirmed wheeze in their GP records, 22.65% were sensitised at this age and 21.70% (133/613) had asthma at age 16 years. Among 578 children with longitudinal data on wheezing to adolescence, 20.93% (121/578) clustered as ETW, 6.75% (39/578) as LOW, 6.75% (48/578) as INT and 8.82% (51/578) as PEW. Table S2 shows u‐EPX levels at age 3 years; u‐EPX was significantly higher among boys and among children who were sensitised.

### 3.2 u‐EPX at age 3 years and wheeze during childhood

Table 1 shows u‐EPX in relation to wheezing during childhood. Children who had any wheeze (parentally reported or doctor‐confirmed), had significantly higher u‐EPX (*p* = .004). Among children with doctor‐confirmed wheeze, there was a strong trend towards u‐EPX being higher among those who had at least one severe exacerbation of wheeze (*p* = .08).

**TABLE 1:** u‐EPX in relation to wheezing during childhood. u‐EPX, urinary‐eosinophil protein X.

		u‐EPX/Cr (μg/mmol)	
Characteristic		GM (95% CI)	*p*‐value
Any wheeze in the first 3 years of life	Yes (*N* = 362)	79.84 (73.87–86.30)	.**004**
	No (*N* = 353)	67.75 (62.51–73.43)	
Severe wheeze exacerbation in the first 3 years of life (*n* = 728)	Yes (*N* = 65)	90.76 (74.65–110.34)	.**019**
	No (*N* = 663)	71.10 (62.47–80.92)	
Severe wheeze exacerbation in the first 3 years of life among children with doctor‐confirmed wheeze (*n* = 293)	Yes (*N* = 64)	90.16 (2.21)	.083
	No (*N* = 229)	74.84 (2.11)	
Wheeze phenotype (cluster)^a^			
NWZ	*N* = 319	64.95 (59.49–70.92)	.**003**
ETW	*N* = 121	77.26 (66.87–89.27)	
LOW	*N* = 39	78.12 (61.81–98.75)	
INT	*N* = 48	92.23 (74.85–113.630)	
PEW	*N* = 51	89.25 (73.02–109.08)	

*Note*: *p*‐values <.05 in bold.

Abbreviations: GM, geometric mean; u‐EPX, urinary‐eosinophil protein X; 95% CI, 95% confidence interval.

^a^ Never wheeze (NWZ); (2) early transient (ETW); (3) late‐onset (LOW); (4) persistent (PEW); and (5) intermittent (INT) wheeze.

#### 3.2.1 Wheeze clusters (phenotypes)^25^

u‐EPX differed significantly between different wheeze phenotypes (*p* = .003, ANOVA; Table 1). The u‐EPX levels were significantly higher among ETW, INT and PEW, compared to NWZ, with numerically highest values among clusters characterised by the persistence of symptoms (INT and PEW; Figure S2A). After the multiple comparison correction (Bonferroni), the difference which remained statistically significant was between NWZ and INT [mean difference (95% CI) −0.35 (−0.69–−0.01); *p* = .038] (Figure S2B).

**FIGURE 1:**
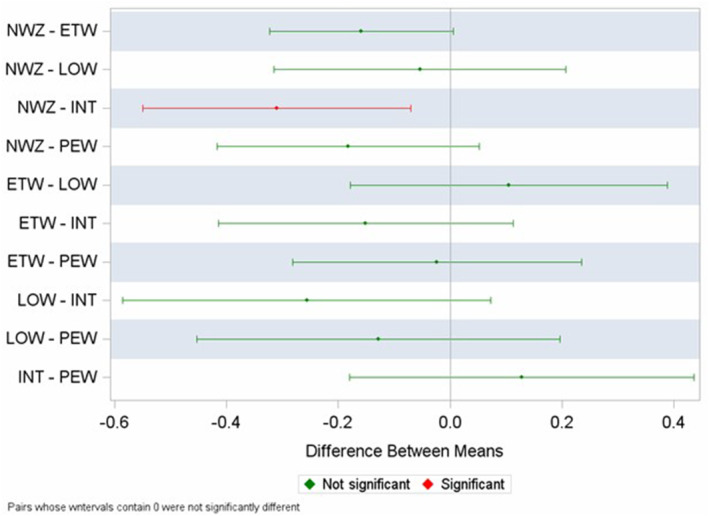
Mean log u‐EPX differences among wheeze phenotypes (NWZ: never wheeze; ETW: early transient wheeze; LOW: late‐onset wheeze; INT: intermittent wheeze; PEW: persistent wheeze). Multivariable model: adjustments were made for sex, maternal sensitisation and sensitisation at age 3 (whole population). u‐EPX, urinary‐eosinophil protein X.

Figure 1 shows the pairwise mean differences of log u‐EPX between different wheeze phenotypes derived from a multivariable model after adjustment for sex, maternal sensitisation and child's sensitisation at age 3 years. U‐EPX differed significantly between the NWZ and INT clusters [mean difference (95% CI) −0.31 (−0.55–−0.07); *p* = .011].

### 3.3 u‐EPX at age 3 years and asthma in adolescence

Univariate associates of asthma diagnosis at age 16 years in the whole population are shown in Table S3. In the univariate analysis, u‐EPX levels were significantly higher among participants with asthma at age 16 years [GM (SD): 95.35 (1.99) vs. 65.82 (2.20), *p* < .001]. A similar trend was observed among those with doctor‐confirmed wheeze in the first 3 years (Table S4).

Results of the multivariable model including u‐EPX at age 3, gestational age, sex, maternal asthma, maternal smoking, maternal allergic sensitisation, child's sensitisation at age 3 years and dog ownership at age 3 years are shown in Table S5. In this model, the association between u‐EPX and asthma diagnosis remained significant, in that the increase in the u‐EPX significantly increased the odds of asthma diagnosis at age 16 years [OR (95% CI): 1.69 (1.16–2.45); *p* = .006]. After adding wheeze within the first 3 years of life into the model (Table 2), the association between u‐EPX and asthma diagnosis was borderline significant [OR (95% CI): 1.44 (0.97–2.05); *p* = .07]. Other factors associated with asthma diagnosis in this model were wheeze in the first 3 years of life, maternal and child's sensitisation.

**TABLE 2:** Associates of asthma diagnosis at age 16 years in the whole cohort. Results of the multiple logistic regression model including u‐EPX at age 3, gestational age, sex, maternal asthma, maternal smoking, maternal allergic sensitisation, child's sensitisation at age 3 years, dog ownership at age 3 and wheeze within the first 3 years of life.

		Asthma diagnosis			
Covariate	Level	Odds ratio	95%CI Low	95%CI Up	*p*‐value
Log u‐EPX at age 3 years	One unit increase	1.44	0.97	2.15	**0.071**
Gestational age	One week increase	0.87	0.74	1.03	0.106
Sex	Female	1.85	0.99	3.44	**0.053**
Maternal asthma	Yes	1.08	0.52	2.23	0.841
Maternal smoking	Yes	1.58	0.57	4.34	0.380
Maternal allergic sensitisation	Yes	2.35	1.20	4.60	**0.013**
Child's sensitisation at age 3 years	Yes	4.61	2.38	8.91	**<.001**
Dog owner, age 3	Yes	1.67	0.71	3.93	0.241
Wheeze ever, age 3	Yes	10.09	5.34	19.03	**<.001**

*Note*: *p*‐value <.1 are reported in bold.

In the multivariable model among children with doctor‐confirmed wheeze in the first 3 years of life (*n* = 293, Table 3), the only two factors that were significantly associated with asthma diagnosis at age 16 years were u‐EPX and allergic sensitisation at age 3, where an increase in u‐EPX significantly increased the odds of asthma [OR (95% CI): 2.3 (1.16–4.37); *p* = .017]. A strong trend was observed for maternal smoking [3.79 (0.93–15.38); *p* = .06].

**TABLE 3:** Associates of asthma diagnosis at age 16 years among children with doctor‐confirmed wheeze: Results of the multiple logistic regression model including u‐EPX at age 3, gestational age, sex, maternal asthma, maternal smoking, maternal allergic sensitisation, child's sensitisation at age 3 years and dog ownership at age 3.

		Asthma diagnosis			
Covariates	Level	Odds Ratio	95%CI Low	95%CI Up	*p*‐value
Log(e) u‐EPX at age 3	One unit increase	2.25	1.16	4.37	**0.017**
Gestational age	One week increase	0.86	0.70	1.06	0.166
Sex	Female	0.86	0.35	2.13	0.748
Maternal asthma	Yes	0.68	0.22	2.15	0.513
Maternal smoking	Yes	3.79	0.93	15.38	**0.062**
Maternal allergic sensitisation	Yes	2.10	0.83	5.30	0.118
Child's sensitisation at age 3	Yes	5.27	2.06	13.45	**<.001**
Dog ownership at age 3	Yes	2.52	0.69	9.26	0.164

*Note*: *p*‐value <.1 are reported in bold.

### 3.4 Prediction of asthma in adolescence

#### 3.4.1 Whole population

We proceeded to determine the discriminative performance on the u‐EPX using the area under the receiver operating characteristics curve (AUC) for all study participants. u‐EPX alone demonstrated moderate performance in predicting asthma diagnosis at age 16 years (AUC 0.65; Figure S3). The optimal cut‐off point was 97 μg/mmol and the corresponding sensitivity, specificity, PPV, NPV and harmonic mean of precision and recall (F1) were 53.3%, 73.5%, 30.6%, 87.7% and 0.57, respectively.

We then determined the discriminative performance of models which included several predictors (sex, u‐EPX, sensitisation and wheeze) using the AUC (Table 4). In the whole cohort, the best performing classification model included sex, u‐EPX, sensitisation and wheeze (AUC = 0.82, 95% CI: 0.76–0.88). u‐EPX and allergic sensitisation alone had similar predictive power (AUC = 0.64, 95% CI: 0.58–0.71) and (AUC = 0.65, 95% CI 0.60–0.71).

**TABLE 4:** Area under the ROC curve with 95% CI for predicting asthma diagnosis at age 16 in the whole population and among children with doctor‐confirmed wheeze in the first 3 years of life.

	AUC (95% CI)^a^	Sensitivity	Specificity	PPV^b^	NPV^c^	F1^d^
Whole population						
Sex, u‐EPX, sensitisation and wheeze	0.82 (0.76–0.88)	0.78	0.80	0.46	0.94	0.58
Sex, u‐EPX and sensitisation	0.70 (0.64–0.76)	0.55	0.79	0.37	0.79	0.44
Sex, u‐EPX and wheeze	0.79 (0.74–0.85)	0.68	0.80	0.43	0.92	0.53
Sex, sensitisation and wheeze	0.81 (0.75–0.86)	0.82	0.72	0.42	0.94	0.55
Sensitised at age 3	0.65 (0.60–0.71)	0.49	0.83	0.42	0.86	0.45
u‐EPX	0.64 (0.58–0.71)	0.53	0.74	0.31	0.88	0.39
Wheeze ever age 3	0.75 (0.69–0.80)	0.76	0.73	0.44	0.91	0.55
Children with doctor‐confirmed wheeze						
Sex, gestational age, maternal sensitisation, u‐EPX, sensitised at age 3	0.76 (0.67–0.85)	0.70	0.70	0.53	0.83	0.60
u‐EPX, sensitised at age 3	0.73 (0.63–0.82)	0.58	0.77	0.56	0.79	0.57
Sensitised at age 3	0.67 (0.59–0.76)	0.48	0.85	0.62	0.77	0.54
u‐EPX	0.63 (0.52–0.76)	0.67	0.58	0.44	0.78	0.53

^a^ 95% confidence intervals.

^b^ Positive predictive value.

^c^ Negative predictive value.

^d^ F‐ score.

#### 3.4.2 Among children with confirmed wheeze in the first 3 years of life

**FIGURE 2:**
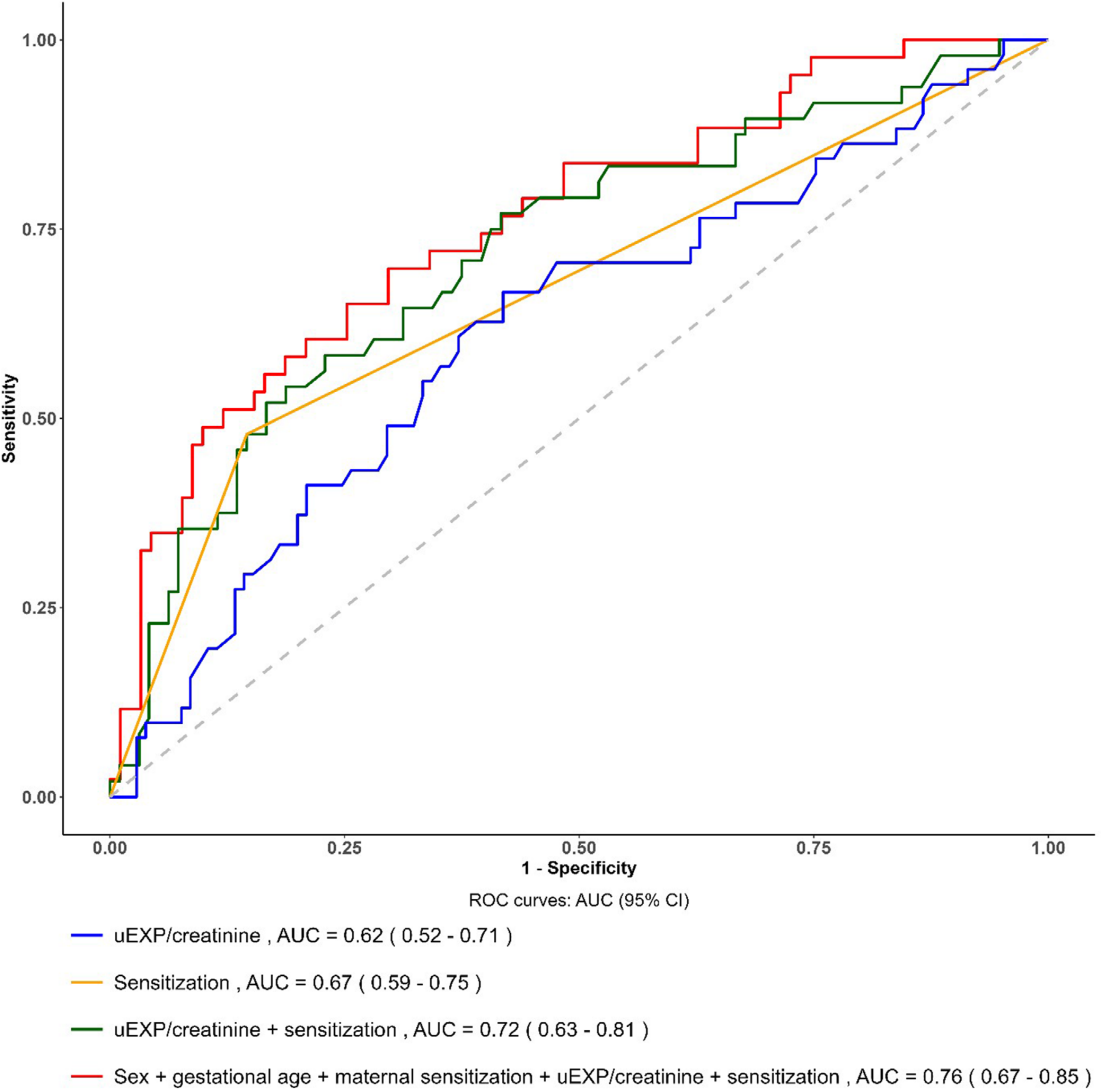
ROC curves for predicting asthma diagnosis at age 16 years among children with physician‐confirmed wheeze at age 3 years. ROC curves, receiver operating characteristic curves.

Figure 2 shows the AUC with 95% CIs for predicting asthma diagnosis at age 16 among children with doctor‐confirmed wheeze in the first 3 years. The best performing classification model included sex, u‐EPX, gestational age, maternal sensitisation and child's sensitisation at age 3 years (AUC: 0.76, 95% CI: 0.67–0.85). The corresponding sensitivity, specificity, PPV, NPV and F1 were 0.70. 0.70. 0.53, 0.83 and 0.60, respectively (Table 4). These values were slightly higher than the model with u‐EPX and child's sensitisation (AUC: 0.73, 95% CI: 0.63–0.82, sensitivity: 0.58, specificity: 0.77, PPV: 0.56, NPV: 0.79 and F1: 0.57). Similarly, sensitisation alone had a marginally higher AUC (95% CI) than u‐EPX alone: 0.67 (0.59–0.76) versus 0.63 (0.52–0.76). The corresponding sensitivity, specificity, PPV, NPV and F1 were 0.48. 0.85. 0.62, 0.77 and 0.54 versus 0.67. 0.58, 0.44, 0.78 and 0.53, respectively. Figure S4 shows the AUC with 95% CIs for predicting asthma diagnosis at age 16 among children with doctor‐confirmed wheeze in the first 3 years using u‐EPX as a binary variable based on the optimal cut‐off point of 97 μg/mmol. The AUC values decreased slightly compared to the model with u‐EPX as a continuous variable.

## 4 DISCUSSION

Several studies to date reported the association of higher u‐EPX levels with childhood asthma (reviewed in^27^), but most were relatively small and cross‐sectional.^27^ Results of the systematic review and meta‐analysis on the use and interpretation of u‐EPX in clinical practice indicated that the potential role of u‐EPX in diagnosing future asthma in children should be investigated in a population‐based prospective cohort study.^27^ In our unselected birth cohort study, we have shown a relationship between u‐EPX levels at age 3 years with asthma at age 16 years, among children who had doctor‐confirmed wheeze within the first 3 years of life but not at the population level. Furthermore, to the best of our knowledge, this is the first study to show differences in the u‐EPX levels in early life between different wheeze phenotypes (defined using information collected from infancy to adolescence). The best performing classification model for asthma prediction among children with doctor‐confirmed wheeze in the first 3 years included child's u‐EPX and sensitisation at age 3 years, sex, gestational age and maternal allergic sensitisation. It is worth noting that both in the whole population and among children with physician‐confirmed wheeze in the first 3 years of life, u‐EPX had almost identical discriminative ability to that of early‐life allergic sensitisation, which has long been considered as one of the strongest associates of wheeze persistence and subsequent asthma diagnosis.^28^ Importantly, u‐EPX is non‐invasive and easily measured in urine, making it potentially more acceptable for young children and their parents and carers compared to more invasive markers such as skin prick tests and measurement of specific serum IgE.

### 4.1 Strengths and limitations

The design of our study gave us an opportunity to look at the whole population data, which is useful in ascertaining associates and may provide pointers towards mechanisms. Importantly, we could also address the issue of how to predict wheeze persistence and future asthma diagnosis among children whose parents/carers attended consultation with their primary care physicians for their child's wheezing illness, which is relevant to clinical practice. This makes the conclusions applicable to the primary care setting.

There are several limitations to our study. The analyses were carried out in the population of predominantly European descent, living in the United Kingdom. Previous studies have shown that profiles of atopic diseases are heterogeneous between countries,^29^, ^30^ and racial/ancestry differences in outcomes such as allergic sensitisation have also been described.^31^, ^32^ We also acknowledge the lack of external validation; however, we could not find another population‐based study with information on both u‐EPX in early‐life and long‐term outcomes. Therefore, further studies are needed to validate our findings in different populations and settings. Specifically, more cohort studies involving mixed ethnicity (especially the black African population) are needed. Until such studies are conducted, we advocate a cautious interpretation of our results, which should be viewed predominantly as a proof‐of concept study. There were also some differences between the included and excluded participants in both predictors and outcomes, but this is unlikely to have a major impact on our findings.

Some of the analyses in relation to wheeze phenotypes were carried out in relatively small subgroups and may therefore not be sufficiently powered to detect differences between the groups and we cannot exclude a possibility of a Type II error. However, our sample size is considerably larger than most previous studies.^27^

We used phenotype of asthma diagnosis at age 16 as a primary outcome for prediction analyses. However, it is likely that such defined asthma diagnosis is heterogeneous^33^ and that studies predicting specific, more homogenous subtypes of asthma are required.^34^ This is highlighted by studies which have shown that a significant proportion of children with persistent wheeze may not have asthma diagnosis.^25^ A recent cluster analysis has shown that early‐life wheeze comprises several distinct clusters with differing relationships to later childhood asthma diagnosis, but that even in the clusters such as infantile‐onset atopic wheeze and early childhood‐onset atopic wheeze, in which one would intuitively expect wheeze persistence and subsequent asthma diagnosis, less than 50% children had asthma diagnosis in school age.^35^

Given the heterogeneity and complexity of the mechanisms which increase the risk (or protect) against the development/persistence of wheezing disorders/asthma, multiple algorithms are likely necessary to predict with enough confidence the persistence of wheeze in children with early wheezing.

### 4.2 Interpretation

Other studies have found higher levels of u‐EPX in children with asthma compared to controls.^36^, ^37^, ^38^ However, most previous studies were cross‐sectional and some were done during the acute episodes of wheezing (e.g. comparing symptomatic patients with asthma with controls^39^; reviewed in^27^). For example, Oymar et al in a study of 32 children (mean age 22 months) found higher u‐EPX in those who were hospitalised with acute wheeze compared with controls (120 vs. 60 μg/mmol).^40^ These values are higher than u‐EPX among children with wheeze in the current study (80.31 μg/mmol), likely because children in our study were assessed when they were symptom free. U‐EPX among healthy children in our population was very similar to those reported in other studies.^27^

In our study, children in the early transient, intermittent and persistent classes (but not those with late‐onset wheeze) had higher u‐EPX than those without wheeze. This suggests that mechanisms associated with eosinophil activation which are associated with late‐onset wheezing may develop later during childhood (the onset of wheezing among those with LOW was after age 8 years^25^). In addition, the overlap in the u‐EPX of the different clusters is a pointer to the heterogeneity within the different wheeze phenotypes. After adjustments for sex, maternal sensitisation and child's sensitisation at age 3, the pairwise difference which remained statistically significant was between the intermittent and no wheeze phenotypes/clusters. This is consistent with observations by Henderson et al. that intermittent wheeze has the strongest associations with allergic sensitisation compared to other wheeze phenotypes.^41^

This associations of sensitisation, male sex and wheeze with higher u‐EPX has been shown in other studies.^18^, ^21^, ^42^ Sex difference in u‐EPX levels remains unexplained. Although the effect of atopy and wheeze (both more common among males) cannot be excluded in the association studies, the effect of u‐EPX on airway symptoms was independent of sex and sensitisation. Males also have impaired innate anti‐viral immunity,^43^ which may contribute to the disruption of epithelial barrier and eosinophil activation independently of sensitisation.

Among children with preschool wheezing, the key area of unmet need for parents and carers is the long‐term prognosis.^1^ Limited number of previous studies addressed the potential value of u‐EPX in asthma prediction. For example, u‐EPX in young children who were hospitalised with acute asthma was associated with the persistence of atopic asthma 2 years later.^40^ In contrast, the same authors have reported in a study of 105 children hospitalised for wheezing during the first year of life that u‐EPX was not a predictive factor for recurrent wheezing.^18^ Chawes et al have shown in the COPSAC cohort that u‐EPX in asymptomatic neonates is associated with later development of allergic sensitisation, nasal eosinophilia and eczema, but not asthma by age 6 years.^44^ In our study, u‐EPX at age 3 years was an independent predictor for asthma at age 16 years and its discriminative ability was moderate (AUC = 0.65). We identified the value of 97 μg/mmol as the most suitable cut off for the prediction of asthma. It is of note that u‐EPX and allergic sensitisation ascertained by SPTs had almost identical predictive power (AUC 0.64 vs. 0.65), but were independent predictors of asthma in multivariable models, suggesting that markers of eosinophil activation add additional valuable information to that of allergic sensitisation.

We did not aim to derive another asthma prediction score, but to ascertain whether u‐EPX as a non‐invasive marker easily measured in urine may be useful in this context. In the whole population, the logistic regression‐based model using sex, u‐EPX, sensitisation and wheeze had the best discriminative power (AUC = 0.82). This is comparable to the discriminative ability of the Childhood Asthma Prediction at Preschool age (CAPP) model developed using machine learning (ML) methods (AUC = 0.82), which was tested in the same population.^8^ A later publication on API as a diagnostic tool for asthma in the general population showed that API has an overall accuracy of 83.6%.^45^ However, the follow‐up period was relatively short (30 months), compared to 13 years in our study.

Similarly, among preschool children with physician‐confirmed wheeze (i.e. those in whom their doctors would be asked questions about the likelihood of long‐term prognosis), the regression‐based model using sex, gestational age, u‐EPX, sensitisation and maternal atopy had the best discriminative power (AUC = 0.76; sensitivity and specificity of 0.7), which is comparable to the MAAS CAPP model for the high‐risk group (AUC = 0.79) derived from ML methods^8^ and not dissimilar to the Pediatric Asthma Risk Score (PARS).^46^, ^47^

In conclusion, u‐EPX, a non‐invasive and cost‐effective test measured in early childhood, may be an additional useful non‐invasive marker for asthma prediction in adolescence. It is likely that such test would be more acceptable to parents compared to more invasive and more expensive tests such as SPTs or blood IgEs.

## AUTHOR CONTRIBUTIONS

**Iso Precious Oloyede:** Formal analysis; writing – original draft; writing – review and editing. **Anhar Ullah:** Formal analysis; writing – original draft; writing – review and editing. **Clare S. Murray:** Conceptualization; methodology; writing – review and editing; writing – original draft. **Sara Fontanella:** Methodology; formal analysis; writing – review and editing; writing – original draft. **Angela Simpson:** Conceptualization; methodology; funding acquisition; project administration; writing – review and editing; supervision. **Adnan Custovic:** Conceptualization; methodology; investigation; formal analysis; supervision; funding acquisition; writing – original draft; writing – review and editing.

## FUNDING INFORMATION

MAAS was supported by the Asthma UK Grants No 301 (1995–1998), No 362 (1998–2001), No 01/012 (2001–2004), No 04/014 (2004–2007), BMA James Trust (2005) and the JP Moulton Charitable Foundation (2004–2016), The North West Lung Centre Charity (1997–present) and the Medical Research Council (MRC) grant MR/L012693/1 (2014–2018).

Infrastructure support for this research was provided by the Imperial and Manchester NIHR Imperial Biomedical Research Centres (BRCs). The views expressed are those of the author(s) and not necessarily those of the NIHR or the Department of Health and Social Care.

## CONFLICT OF INTEREST STATEMENT

Dr. Custovic reports personal fees from Sanofi, La Roche‐Posay, Reacta Healthcare and outside the submitted work. AS reports lecture fees from Thermo Fisher Scientific. Other authors have no competing interests to declare.

### PEER REVIEW

The peer review history for this article is available at https://www.webofscience.com/api/gateway/wos/peer‐review/10.1111/pai.70013.

## Supporting information


Data S1.

## References

[1] Makrinioti H , Fainardi V , Bonnelykke K , et al. European Respiratory Society statement on preschool wheezing disorders: updated definitions, knowledge gaps, and proposed future research directions. Eur Respir J. 2024;64:2400624. 38843917 10.1183/13993003.00624-2024

[2] Salehian S , Fleming L , Saglani S , Custovic A . Phenotype and endotype based treatment of preschool wheeze. Expert Rev Respir Med. 2023;17(10):853‐864. 37873657 10.1080/17476348.2023.2271832

[3] Oksel C , Granell R , Haider S , et al. Distinguishing wheezing phenotypes from infancy to adolescence. A pooled analysis of five birth cohorts. Ann Am Thorac Soc. 2019;16(7):868‐876. 30888842 10.1513/AnnalsATS.201811-837OC PMC6600832

[4] Koefoed HJL , Vonk JM , Koppelman GH . Predicting the course of asthma from childhood until early adulthood. Curr Opin Allergy Clin Immunol. 2022;22(2):115‐122. 35197433 10.1097/ACI.0000000000000810 PMC8915994

[5] Castro‐Rodriguez JA , Holberg CJ , Wright AL , Martinez FD . A clinical index to define risk of asthma in young children with recurrent wheezing. Am J Respir Crit Care Med. 2000;162(4):1403‐1406. 11029352 10.1164/ajrccm.162.4.9912111

[6] Pescatore AM , Dogaru CM , Duembgen L , et al. A simple asthma prediction tool for preschool children with wheeze or cough. J Allergy Clin Immunol. 2014;133(1):111‐118.e13. 23891353 10.1016/j.jaci.2013.06.002

[7] Colicino S , Munblit D , Minelli C , Custovic A , Cullinan P . Validation of childhood asthma predictive tools: a systematic review. Clin Exp Allergy. 2019;49(4):410‐418. 30657220 10.1111/cea.13336

[8] Kothalawala DM , Murray CS , Simpson A , et al. Development of childhood asthma prediction models using machine learning approaches. Clin Transl Allergy. 2021;11(9):e12076. 34841728 10.1002/clt2.12076 PMC9815427

[9] Kothalawala DM , Kadalayil L , Curtin JA , et al. Integration of genomic risk scores to improve the prediction of childhood asthma diagnosis. J Pers Med. 2022;12(1). 10.3390/jpm12010075 PMC8777841 35055391

[10] Farhan AJ , Kothalawala DM , Kurukulaaratchy RJ , et al. Prediction of adult asthma risk in early childhood using novel adult asthma predictive risk scores. Allergy. 2023;78(11):2969‐2979. 37661293 10.1111/all.15876 PMC10840748

[11] Lowe L , Murray CS , Martin L , et al. Reported versus confirmed wheeze and lung function in early life. Arch Dis Child. 2004;89(6):540‐543. 15155399 10.1136/adc.2003.038539 PMC1719938

[12] Sly PD , Boner AL , Bjorksten B , et al. Early identification of atopy in the prediction of persistent asthma in children. Lancet. 2008;372(9643):1100‐1106. 18805338 10.1016/S0140-6736(08)61451-8 PMC4440493

[13] Bannier MA , van de Kant KD , Jobsis Q , Dompeling E . Biomarkers to predict asthma in wheezing preschool children. Clin Exp Allergy. 2015;45(6):1040‐1050. 25409553 10.1111/cea.12460

[14] Kothalawala DM , Kadalayil L , Weiss VBN , et al. Prediction models for childhood asthma: a systematic review. Pediatr Allergy Immunol. 2020;31(6):616‐627. 32181536 10.1111/pai.13247

[15] Castro‐Rodriguez JA . The necessity of having asthma predictive scores in children. J Allergy Clin Immunol. 2013;132(6):1311‐1313. 24172768 10.1016/j.jaci.2013.09.006

[16] Custovic D , Fontanella S , Custovic A . Understanding progression from pre‐school wheezing to school‐age asthma: can modern data approaches help? Pediatr Allergy Immunol. 2023;34(12):e14062. 38146116 10.1111/pai.14062

[17] Chedevergne F , Le Bourgeois M , de Blic J , Scheinmann P . The role of inflammation in childhood asthma. Arch Dis Child. 2000;82:S6‐S9. 10.1136/adc.82.suppl_2.ii6 PMC1765084 10833470

[18] Oymar K , Havnen J , Halvorsen T , Bjerknes R . Eosinophil counts and urinary eosinophil protein X in children hospitalized for wheezing during the first year of life: prediction of recurrent wheezing. Acta Paediatr. 2001;90(8):843‐849. 11529528

[19] Custovic A , Simpson BM , Murray CS , Lowe L , Woodcock A , The NAC Manchester Asthma and Allergy Study Group . The National Asthma Campaign Manchester Asthma and allergy study. Pediatr Allergy Immunol. 2002;13(s15):32‐37. 12688622 10.1034/j.1399-3038.13.s.15.3.x

[20] Semic‐Jusufagic A , Belgrave D , Pickles A , et al. Assessing the association of early life antibiotic prescription with asthma exacerbations, impaired antiviral immunity, and genetic variants in 17q21: a population‐based birth cohort study. Lancet Respir Med. 2014;2(8):621‐630. 24835835 10.1016/S2213-2600(14)70096-7

[21] Gore C , Peterson CGB , Kissen P , et al. Urinary eosinophilic protein X, atopy, and symptoms suggestive of allergic disease at 3 years of age. J Allergy Clin Immunol. 2003;112(4):702‐708. 14564347 10.1016/s0091-6749(03)01886-4

[22] Kristjánsson S , Strannegård IL , Strannegård O , Peterson C , Enander I , Wennergren G . Urinary eosinophil protein X in children with atopic asthma: a useful marker of antiinflammatory treatment. J Allergy Clin Immunol. 1996;97(6):1179‐1187. 8648010 10.1016/s0091-6749(96)70182-3

[23] Reddel HK , Taylor DR , Bateman ED , et al. An official American Thoracic Society/European Respiratory Society statement: asthma control and exacerbations: standardizing endpoints for clinical asthma trials and clinical practice. Am J Respir Crit Care Med. 2009;180(1):59‐99. 19535666 10.1164/rccm.200801-060ST

[24] Pinart M , Benet M , Annesi‐Maesano I , et al. Comorbidity of eczema, rhinitis, and asthma in IgE‐sensitised and non‐IgE‐sensitised children in MeDALL: a population‐based cohort study. Lancet Respir Med. 2014;2(2):131‐140. 24503268 10.1016/S2213-2600(13)70277-7

[25] Haider S , Granell R , Curtin J , et al. Modeling wheezing spells identifies phenotypes with different outcomes and genetic associates. Am J Respir Crit Care Med. 2022;205(8):883‐893. 35050846 10.1164/rccm.202108-1821OC PMC9838626

[26] Youden WJ . Index for rating diagnostic test. Cancer. 1950;3:32‐35. 15405679 10.1002/1097-0142(1950)3:1<32::aid-cncr2820030106>3.0.co;2-3

[27] Klonoff‐Cohen H , Polavarapu M . Eosinophil protein X and childhood asthma: a systematic review and meta‐analysis. Immun Inflamm Dis. 2016;4(2):114‐134. 27957324 10.1002/iid3.104 PMC4879459

[28] Custovic A , Custovic D , Fontanella S . Understanding the heterogeneity of childhood allergic sensitization and its relationship with asthma. Curr Opin Allergy Clin Immunol. 2024;24(2):79‐87. 38359101 10.1097/ACI.0000000000000967 PMC10906203

[29] Dramburg S , Grittner U , Potapova E , et al. Heterogeneity of sensitization profiles and clinical phenotypes among patients with seasonal allergic rhinitis in southern European countries‐the @IT.2020 multicenter study. Allergy. 2024;79(4):908‐923. 38311961 10.1111/all.16029

[30] Kiewiet MBG , Lupinek C , Vrtala S , et al. A molecular sensitization map of European children reveals exposome‐ and climate‐dependent sensitization profiles. Allergy. 2023;78(7):2007‐2018. 36815272 10.1111/all.15689

[31] Wegienka G , Johnson CC , Zoratti E , Havstad S . Racial differences in allergic sensitization: recent findings and future directions. Curr Allergy Asthma Rep. 2013;13(3):255‐261. 23435599 10.1007/s11882-013-0343-2 PMC4888051

[32] Somoza ML , Perez‐Sanchez N , Torres‐Rojas I , et al. Sensitisation to pollen allergens in children and adolescents of different ancestry born and living in the same area. J Asthma Allergy. 2022;15:1359‐1367. 36189188 10.2147/JAA.S370279 PMC9525024

[33] Pavord ID , Beasley R , Agusti A , et al. After asthma: redefining airways diseases. Lancet. 2018;391(10118):350‐400. 28911920 10.1016/S0140-6736(17)30879-6

[34] Custovic A , Siddiqui S , Saglani S . Considering biomarkers in asthma disease severity. J Allergy Clin Immunol. 2022;149(2):480‐487. 34942235 10.1016/j.jaci.2021.11.021

[35] Ngo SY , Venter C , Anderson WC 3rd , et al. Clinical features and later prognosis of replicable early‐life wheeze clusters from two birth cohorts 12 years apart. Pediatr Allergy Immunol. 2023;34(7):e13999. 37492911 10.1111/pai.13999 PMC10372879

[36] Labbé A , Aublet‐Cuvelier B , Jouaville L , et al. Prospective longitudinal study of urinary eosinophil protein X in children with asthma and chronic cough. Pediatr Pulmonol. 2001;31(5):354‐362. 11340681 10.1002/ppul.1058

[37] Kristjánsson S , Wennergren D , Eriksson B , Thórarinsdóttir H , Wennergren G . U‐EPX levels and wheezing in infants and young children with and without RSV bronchiolitis. Respir Med. 2006;100(5):878‐883. 16198099 10.1016/j.rmed.2005.08.013

[38] Lugosi E , Halmerbauer G , Frischer T , Koller D . Urinary eosinophil protein X in relation to disease activity in childhood asthma. Allergy. 1997;52(5):584‐588. 9201373 10.1111/j.1398-9995.1997.tb02605.x

[39] Hoekstra M , Hovenga H , Gerritsen J , Kauffman H . Eosinophils and eosinophil‐derived proteins in children with moderate asthma. 1996;9(11):2231‐2235. 10.1183/09031936.96.09112231 8947065

[40] Oymar K . High levels of urinary eosinophil protein X in young asthmatic children predict persistent atopic asthma. Pediatr Allergy Immunol. 2001;12(6):312‐317. 11846868 10.1034/j.1399-3038.2001.0o080.x

[41] Henderson J , Granell R , Heron J , et al. Associations of wheezing phenotypes in the first 6 years of life with atopy, lung function and airway responsiveness in mid‐childhood. Thorax. 2008;63(11):974‐980. 18678704 10.1136/thx.2007.093187 PMC2582336

[42] Tauber E , Halmerbauer G , Frischer T , et al. Urinary eosinophil protein X in children: the relationship toasthma and atopy and normal values. Allergy. 2000;55(7):647‐652. 10921464 10.1034/j.1398-9995.2000.00528.x

[43] Regis E , Fontanella S , Lin L , et al. Sex differences in innate anti‐viral immune responses to respiratory viruses and in their clinical outcomes in a birth cohort study. Sci Rep. 2021;11(1):23741. 34887467 10.1038/s41598-021-03044-x PMC8660814

[44] Chawes BL , Bønnelykke K , Bisgaard H . Elevated eosinophil protein X in urine from healthy neonates precedes development of atopy in the first 6 years of life. Am J Respir Crit Care Med. 2011;184(6):656‐661. 21680952 10.1164/rccm.201101-0111OC

[45] Castro‐Rodriguez JA , Forno E , Padilla O , Casanello P , Krause BJ , Borzutzky A . The asthma predictive index as a surrogate diagnostic tool in preschoolers: analysis of a longitudinal birth cohort. Pediatr Pulmonol. 2021;56(10):3183‐3188. 34320686 10.1002/ppul.25592 PMC10772975

[46] Biagini Myers JM , Schauberger E , He H , et al. A pediatric asthma risk score to better predict asthma development in young children. J Allergy Clin Immunol. 2019;143(5):1803‐1810 e2. 30554722 10.1016/j.jaci.2018.09.037 PMC6504569

[47] Biagini JM , Martin LJ , He H , et al. Performance of the pediatric asthma risk score across diverse populations. NEJM Evid. 2023;2(10):EVIDoa2300026. 38320177 10.1056/EVIDoa2300026 PMC11697972

