# Supplementary material for: Association of urinary eosinophilic protein X at age 3 years and subsequent persistence of wheezing and asthma diagnosis in adolescence
Source: Pediatr Allergy Immunol. 2024 Dec 4;35(12):e70013. doi: 10.1111/pai.70013 (PMC11616470; doi:10.1111/pai.70013)
Supplement: Supplementary file 1 — Data S1. [file PAI-35-e70013-s001.docx]

**Association of urinary eosinophilic protein X at age 3 years and subsequent persistence of wheezing and asthma diagnosis in adolescence**

Iso Precious Oloyede MD MRes^1^, Anhar Ullah^1^, Clare S Murray MD^2^, Sara Fontanella^1^, Angela Simpson MD PhD^2^*, Adnan Custovic MD PhD FMedSci*^1^

^1^National Heart and Lung Institute, Imperial College London, UK

^2^Division of Infection, Immunity and Respiratory Medicine, Faculty of Biology, Medicine and Health, Manchester Academic Health Sciences Centre, University of Manchester and University Hospital of South Manchester NHS Foundation Trust, Manchester, UK

*Equal contribution, joint senior authors

**SUPPLEMENTARY APPENDIX**

**METHODS**

**SUPPLEMENTARY METHODS**

**Data sources: description of cohort**

The Manchester Asthma and Allergy Study (MAAS) MAAS is an unselected birth cohort study established in 1995 in Manchester, UK ^1^. It consists of a mixed urban-rural population within 50 square miles of South Manchester and Cheshire, located within the maternity catchment area of Wythenshawe and Stepping Hill Hospitals. All pregnant women were screened for eligibility at antenatal visits (8-10th week of pregnancy). Of the 1499 couples who met the inclusion criteria (≤10 weeks of pregnancy, maternal age ≥18 years, and questionnaire and skin prick data test available for both parents), 288 declined to take part in the study and 27 were lost to follow-up between recruitment and the birth of a child. A total of 1184 children were born into the study between February 1996 and April 1998. They were followed prospectively for 20 years to date and attended follow-up clinics for assessments, which included lung function measurements, skin prick testing, biological samples (serum, plasma and urine), and questionnaire data collection. The study was approved by the North West – Greater Manchester East Research Ethics Committee.

**Definitions of variables (outcomes, demographic, and exposures)**

**Any wheeze at age 3 years:** Parentally-reported wheeze and/or physician confirmed wheeze in the first three years of life.

**Maternal asthma:** Defined based on the responses given to the question “Do you have or have you ever been told you to have asthma?” administered during pregnancy.

**Maternal smoking:** Defined based on the response given to the question “Do you smoke”, administered during pregnancy.

**Maternal sensitisation (SPT):** Defined as mother being sensitised to at least one allergen using SPT, with a mean wheal diameter of at least 3mm larger than that elicited by the negative control.

**Paternal sensitisation (SPT):** Defined as fathers being sensitised to at least one allergen using SPT, with a mean wheal diameter of at least 3mm larger than that elicited by the negative control.

**BMI (Kg/m2) at birth:** Weight and height were measured at birth

**BMI (Kg/m2) at age 3:** Weight and height were measured at age 3 clinic visit.

**Gestational age:** Age in weeks, from the date of the mother’s last menstrual period to delivery.

**Cat owner at age 3:** Defined based on the response to the question “Do you own a cat? administered at the age 3 clinic visit.

**Dog owner at age 3:** Defined based on the response to the question “Do you own a dog? administered at the age 3 clinic visit.

**Measurement of Urinary Eosinophilic Protein X**

Urine samples were collected at the age three years, divided in aliquots, and frozen at -20oC. The urine samples were analysed in duplicate for EPX by means of a sensitive and specific Radioimmunoassay (RIA) (Pharmacia Diagnostics AB, Uppsala Sweden). Immediately before assessment of EPX, the urine was diluted 11 times in a phosphate buffer, pH 7.4, containing 0.15 mol/L Sodium Chloride (NaCl), 1% Bovine Serum Albumin (BSA), 0.1% Tween-20, 10 mmol/L Sodium ethylenediaminetetraacetic acid (Na2-EDTA), and 0.2% N-cetyl-N-N-N-trimethylammonium bromide.^2,3^ The detection limit was less than 3 μg/L, and the coefficients of variation on the basis of three control samples analysed in four replicates on 25 occasions were between 4.1% and 5.8% (within assays) and between 2.7% and 3.7% (between assays). Mean recoveries after addition of 3 purified EPX concentrations to 3 normal urine samples were 96%, 103%, and 105% of expected value.^4^

Due to the absence of a total urine volume, the extent of dilution of urine in the kidneys was determined by measuring the urine creatinine (Cr) using the alkaline picrate method (Jaffé reaction; HiCo Creatinine; Boehringer Mannheim GmbH, Mannheim, Germany). The results are presented as the U-EPX/creatinine ratio (in micrograms per millimole).

**RESULTS**

**Figure E1:** Consort diagram describing the participant flow


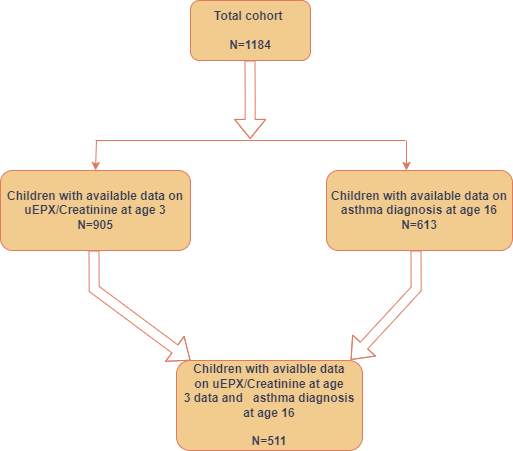


**Table E1:** Demographic and clinical characteristics of study participants. (Whole population)

| **Factors** |  |
| --- | --- |
| Male, n/N (%) | 476/905 (52.60) |
| Gestational age, mean (SD) | 39.88 (1.60) |
| BMI at birth, mean (SD) | 12.12 (1.71) |
| Maternal asthma, n/N (%) | 167/905 (18.45) |
| Maternal smoking, n/N (%) | 121/905 (13.37) |
| Maternal sensitisation (SPT), n/N (%) | 533/901 (59.03) |
| Paternal sensitisation (SPT), n/N (%) | 575/902 (63.82) |
| Sensitised at age 3 years, n/N (%) | 190/839 (22.65) |
| Maternal smoking at age 3, n/N (%) | 161/900 (17.89) |
| Cat owner at age 3, n/N (%) | 160/900 (17.78) |
| Dog owner at age 3, n/N (%) | 113/900 (12.56) |
| Wheeze ever at age 3, n/N (%) | 332/903 (36.77) |
| Physician-confirmed wheezer | 293/715 (40.98) |
| Asthma diagnosis at age 16, n/N (%) | 133/613 (21.70) |
| BMI at age 3, mean(SD) | 16.65 (1.38) |
| uEPX/creatinine, GM(SD) | 72.48 (2.14) |
| *Wheeze phenotypes* |  |
| NWZ, n/N (%) | 319 (55.19) |
| ETW, n/N (%) | 121 (20.93) |
| LOW, n/N (%) | 39 (6.75) |
| INT, n/N (%) | 48 (8.30) |
| PEW, n/N (%) | 51 (8.82) |
| N: total number of non-missing data  SD: standard deviation  GM: Geometric mean | |

| **Covariates** | **Level** | **N** | **uEPX/Creatinine, age 3**  **GM (95% CI)** | **P-value** |
| --- | --- | --- | --- | --- |
| Sex | Female | 429 | 65.98 (61.51-70.77) | **<.001** |
|  | Male | 476 | 78.90 (73.57-84.62) |  |
| Maternal asthma ever | No | 738 | 72.80 (68.99-76.82) | 0.952 |
|  | Yes | 167 | 71.10 (62.47-80.92) |  |
| Maternal smoking pregnancy | No | 784 | 71.52 (67.77-75.48) | 0.177 |
|  | Yes | 121 | 79.06 (69.32-90.17) |  |
| Maternal sensitisation | No | 370 | 70.77 (65.60-76.35) | 0.221 |
|  | Yes | 533 | 73.86 (69.13-78.92) |  |
| Paternal sensitisation | No | 326 | 70.95 (65.43-76.93) | 0.539 |
|  | Yes | 575 | 73.48 (68.96-78.30) |  |
| Child’s sensitisation at age 3 years | No | 649 | 66.22 (62.53-70.12) | **<.001** |
|  | Yes | 190 | 93.34 (83.89-103.86) |  |
| Maternal smoking | No | 739 | 71.50 (67.66-75.55) | 0.331 |
|  | Yes | 161 | 77.07 (68.48-86.74) |  |
| Cat owner at age 3 | No | 740 | 73.17 (69.29-77.26) | 0.640 |
|  | Yes | 160 | 69.29 (61.11-78.57) |  |
| Dog owner at age 3 | No | 787 | 71.54 (67.79-75.49) | 0.158 |
|  | Yes | 113 | 79.25 (69.32-90.61) |  |
| GM: Geometric mean  P-value < 0.1 are reported in bold. | | | | |

**Table E2:** uEPX/creatinine at age 3 years in the whole population

**Figure E2:** Pairwise mean log u-EPX differences among wheeze phenotypes (NWZ: Never wheeze; ETW: Early Transient Wheeze; LOW: Late onset wheeze; INT: Intermittent wheeze; PEW: Persistent wheeze) before (Panel A) and after the Bonferroni multiple-comparison correction (Panel B); Whole population.

A)


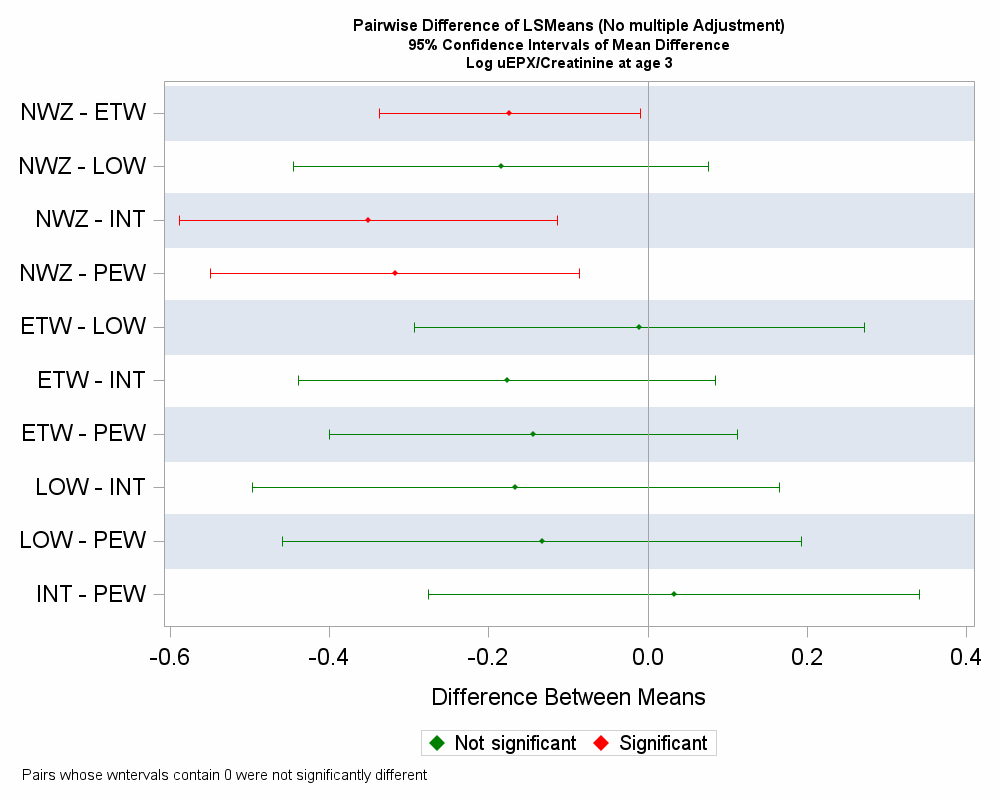

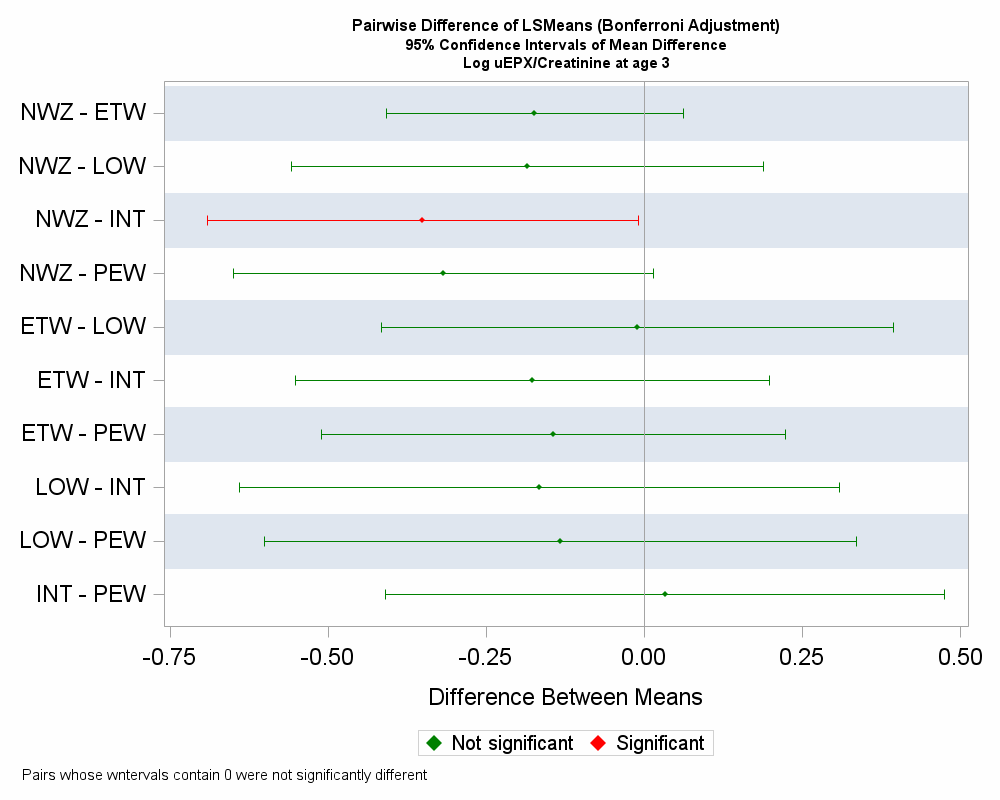


B)

**Table E3:** Univariate associates of asthma diagnosis in the whole cohort

| **Covariates** | **No Asthma diagnosis**  **N=480** | **Asthma diagnosis**  **N=133** | **P-value** |
| --- | --- | --- | --- |
| Male, n (%) | 234 (48.75) | 79 (59.4) | **0.03** |
| Maternal asthma, n (%) | 79 (16.46) | 32 (24.06) | **0.04** |
| Paternal asthma, n (%) | 63 (13.13) | 24 (18.05) | 0.150 |
| Maternal smoking, n (%) | 41 (8.54) | 18 (13.85) | **0.07** |
| Maternal sensitisation, n (%) | 264 (55.11) | 87 (71.9) | **<.001** |
| Paternal sensitisation, n (%) | 305 (63.67) | 80 (67.8) | 0.402 |
| Gestational age, mean (SD) | 39.98 (1.51) | 39.69 (1.94) | **0.088** |
| BMI at birth, mean (SD) | 12.15 (1.42) | 12.23 (2.10) | 0.676 |
| Sensitised at age 3, n (%) | 74 (17.25) | 53 (48.62) | **<.001** |
| Maternal smoking at age 3, n (%) | 57 (12.23) | 16 (13.33) | 0.745 |
| Cat owner at age 3, n (%) | 94 (20.17) | 23 (20) | 0.967 |
| Dog owner at age 3, n (%) | 46 (9.87) | 19 (16.38) | **0.046** |
| Wheeze ever at age 3, n (%) | 113 (23.94) | 90 (72.58) | **<.001** |
| uEPX/Creatinine at age 3, GM(SD) | 65.82 (2.20) | 95.35(1.99) | **<.001** |
| BMI at age 3, mean (SD) | 16.63 (1.34) | 16.72 (1.26) | 0.490 |
| SD: Standard deviation  GM: Geometric mean  P-value < 0.1 are reported in bold. | | | |

**Table E4:** Univariate associates of asthma diagnosis among participants with physician-confirmed wheeze at age 3.

| **Covariate** | **No Asthma diagnosis**  **N=105** | **Asthma diagnosis**  **N=51** | **P-value** |
| --- | --- | --- | --- |
| Male, n (%) | 59 (56.19) | 33 (64.71) | 0.310 |
| Maternal asthma, n (%) | 21 (20) | 11 (21.57) | 0.820 |
| Paternal asthma, n (%) | 18 (17.14) | 7 (13.73) | 0.585 |
| Maternal smoking, n (%) | 6 (5.71) | 7 (13.73) | **0.089** |
| Maternal sensitisation , n (%) | 57 (54.29) | 39 (76.47) | **0.008** |
| Paternal sensitisation, n (%) | 62 (59.05) | 34 (66.67) | 0.359 |
| Gestational age, mean (SD) | 39.93 (1.73) | 39.69 (2.43) | 0.498 |
| BMI at birth, mean (SD) | 12.01 (1.53) | 11.7 (1.41) | 0.248 |
| Sensitised at age 3, n (%) | 14 (14.58) | 23 (47.92) | **<.001** |
| Maternal smoking at age 3, n (%) | 8 (7.62) | 8 (16) | 0.109 |
| Cat owner at age 3, n (%) | 23 (21.9) | 8 (16) | 0.390 |
| Dog owner at age 3, n (%) | 10 (9.52) | 7 (14) | 0.404 |
| uEPX/Creatinine ratio, age 3, GM(SD) | 69.36 (2.09) | 91.47 (2.08) | **0.019** |
| BMI at age 3, mean (SD) | 16.74 (1.36) | 16.7 (1.19) | 0.847 |
| P-value < 0.1 are reported in bold. | | | |

**Table E5:** Associates of asthma diagnosis at age 16 years in the whole cohort.:

Results of the multiple logistic regression model including u-EPX at age 3, gestational age, sex, maternal asthma, maternal smoking, maternal allergic sensitisation, child’s sensitisation at age 3 years and dog ownership at age 3.

|  | | **Asthma diagnosis** | | | |
| --- | --- | --- | --- | --- | --- |
| **Covariates** | **Level** | **Odds Ratio** | **95%CI Low** | **95%CI Up** | **P-value** |
| Log uEPX/Creatinine at age 3 | One unit increase | 1.69 | 1.16 | 2.45 | **0.006** |
| Gestational age | One week increase | 0.83 | 0.71 | 0.97 | **0.019** |
| Sex | Female | 1.31 | 0.76 | 2.27 | 0.334 |
| Maternal asthma | Yes | 1.24 | 0.62 | 2.45 | 0.543 |
| Maternal smoking | Yes | 2.33 | 0.99 | 5.52 | **0.054** |
| Maternal allergic sensitisation | Yes | 1.96 | 1.06 | 3.60 | **0.031** |
| Child’s sensitisation at age 3 | Yes | 3.70 | 2.08 | 6.58 | **<.001** |
| Dog ownership at age 3 | Yes | 2.08 | 0.94 | 4.59 | **0.070** |
| P-value < 0.1 are reported in bold. | | | | | |

**Figure E3:** A ROC curve showing the sensitivity and specificity of uEPX in the prediction of asthma at age 16


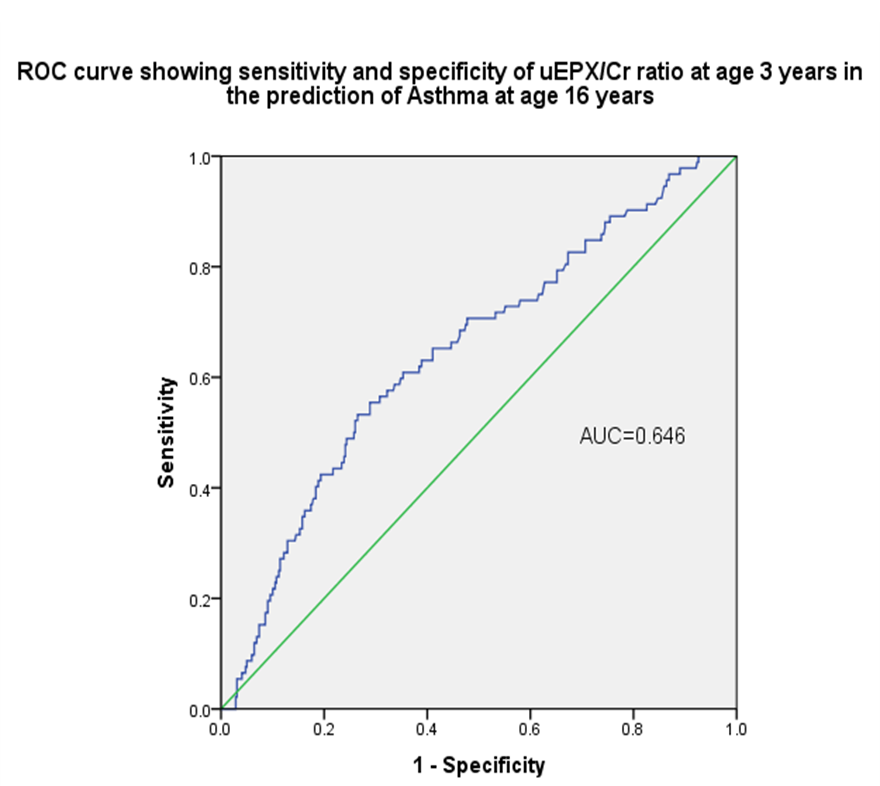


**Figure E4:** ROC curves for predicting asthma diagnosis at age 16 years among children with physician-confirmed wheeze at age 3 years. u-EPX used as binary variable based on the optimal cut-off point of 97µg/mmol.


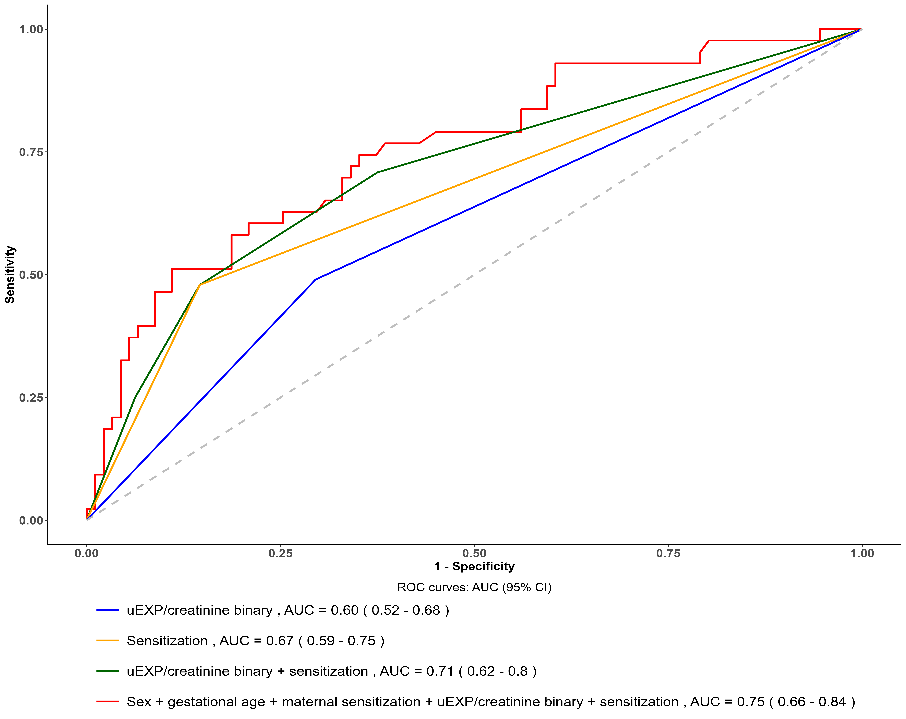


**References**

1. Custovic A, Simpson BM, Murray CS, Lowe L, Woodcock A, Group NMAaAS. The National Asthma Campaign Manchester Asthma and Allergy Study. *Pediatr Allergy Immunol* 2002; **13 Suppl 15**: 32-7.

2. Kristjánsson S, Strannegård IL, Strannegård O, Peterson C, Enander I, Wennergren G. Urinary eosinophil protein X in children with atopic asthma: a useful marker of antiinflammatory treatment. *J Allergy Clin Immunol* 1996; **97**(6): 1179-87.

3. Oymar K, Havnen J, Halvorsen T, Bjerknes R. Eosinophil counts and urinary eosinophil protein X in children hospitalized for wheezing during the first year of life: prediction of recurrent wheezing. *Acta Paediatrica* 2001; **90**(8): 843-9.

4. Gore C, Peterson CGB, Kissen P, et al. Urinary eosinophilic protein X, atopy, and symptoms suggestive of allergic disease at 3 years of age. *Journal of Allergy and Clinical Immunology* 2003; **112**(4): 702-8.
